# The PPARγ2 Pro12Ala variant is protective against progression of nephropathy in people with type 2 diabetes

**DOI:** 10.1186/s12967-015-0448-6

**Published:** 2015-03-12

**Authors:** Emanuela Lapice, Antonella Monticelli, Sergio Cocozza, Michele Pinelli, Sara Cocozza, Dario Bruzzese, Gabriele Riccardi, Olga Vaccaro

**Affiliations:** Department of Clinical Medicine and Surgery, University of Naples Federico II, Via S Pansini 5, Naples, 80131 Italy; Department of Cellular and Molecular Biology and Pathology A. Califano DBPCM, University of Naples Federico II, Via S Pansini 5, Naples, 80131 Italy; IEOS CNR, Via S Pansini 5, Naples, 80131 Italy; Department of Public Health, University of Naples Federico II, Via S Pansini 5, Naples, 80131 Italy

**Keywords:** Nephropathy, PPAR-gamma polymorphism, Type 2 diabetes

## Abstract

**Objective:**

Cross-sectional studies suggest the association between diabetic nephropathy and the PPARγ2 Pro12Ala polymorphism of the peroxisome proliferator-activated receptor γ2 (PPARγ2). Prospective data are limited to microalbuminuria and no information on renal function is available to date. The present study evaluates the association between the Pro12Ala polymorphism of PPARγ2 and the progression of albuminuria and decay in glomerular filtration rate (GFR) in type 2 diabetes.

**Patients and measurements:**

We studied 256 patients with an average 5-year follow-up. Among others, urinary albumin excretion rate (UAER) was measured on spot sample, GFR was estimated with the CKD-EPI Equation.

**Results:**

Baseline UAER and GFR were similar for carriers or non-carriers of the polymorphism. At follow-up no significant changes from baseline were observed for UAER or eGFR in carriers of the Pro12Ala polymorphism whereas a significant increase in UAER [17 (11.3-37.9) versus 24.5 (13.8-49.9) μg/mg, p < 0.006)] and a significant reduction in the eGFR (82.8 ± 14.5 versus 80.3 ± 17.3 ml/min/1.73, m^2^ p = 0.02), were observed in non carriers of the Pro12Ala polymorphism. Progression of nephropathy - defined according to a combined end point of UAER and eGFR- i.e. doubling of baseline UAER to at least 100 μg/mg, or new onset microalbuminuria, or progression from micro to macroalbuminuria, or 25% reduction of eGFR, or annualized eGFR decline >3 ml/min/year - was significantly less frequent in Ala carriers than non carriers (11.4% vs 35.8%; p < 0.01); HR adjusted for baseline age, AER, eGFR, HbA1c, diabetes duration and blood pressure was 0.32 (0.12-0.80).

**Conclusions:**

This study found that among patients with type 2 diabetes, the PPARγ2 Pro12Ala polymorphism is protective against progression of nephropathy and decay of renal function independent of major confounders.

## Introduction

The etiology of diabetic nephropathy is not fully understood; while environmental factors certainly play a major role, the familial aggregation of the disease and the disproportionate prevalence among specific ethnic groups, suggests that genetic factors may influence the risk of the disease [1]. Among others the peroxisome proliferator-activated receptor (PPARγ) gene has been associated with the risk of diabetic nephropathy [2]. In particular the Ala12 variant of the PPAR γ2 isoform has been consistently associated with less insulin resistance and increased resistance to oxidative stress [3,4]. Both conditions have been suggested to contribute to the development and progression of diabetic nephropathy [5-7]. Moreover animal and human studies suggest that the thiazolidinediones, which act as PPARγ2 agonists, may reduce urinary albumin excretion rate (UAER) and may prevent the development of renal injury [8], thus further supporting a role for PPARγ2 in the phenotype diabetic nephropathy. So far genetic association studies investigating the relation of the PPARγ2 Pro12Ala polymorphism with diabetic nephropathy have provided not entirely consistent results [9-17]. Differences in the methods for the assessment of UAER (i.e. timed urine collection versus a spot urine albumin-to-creatinine ratio, single or repeated measures of albuminuria) and different ethnic composition and genetic susceptibility to nephropathy of the populations studied, may partly explain discrepancies in the findings. In addition all these studies were cross sectional and therefore liable to survival bias, which may further contribute to the variability of the results [18,19]. Longitudinal studies would overcome some of these limitations but existing evidence is limited to one study which has shown a lower frequency of new-onset microalbuminuria in people carriers of the PPARγ2 Ala variant [20]. This study was performed within the context of a randomized clinical trial designed to evaluate whether ACE inhibitors and calcium-channel blockers prevent new onset microalbuminuria in a selected group of diabetic people with hypertension, normoalbuminuria and no evidence of renal injury at baseline [20]. Data on renal function were not provided in this study, furthermore, due to selection criteria, it is not known to what extent the findings can be extrapolated to diabetic patients at large, including patients with more advanced renal damage. The present work expands current knowledge by evaluating the association of the Pro12Ala polymorphism of PPARγ2 with the progression of urinary albumin excretion and decay of glomerular filtration rate overtime in a clinic-based sample of patients with type 2 diabetes, representative of the diabetic patients seen in clinical practice.

## Patients and methods

The study participants are 256 males and females with type 2 diabetes consecutively attending the outpatients Diabetes Clinic of the University Hospital for the screening of diabetes complications and with two complete examinations performed at least one year apart between January 1th 2002 and January 1th 2013. The longest available follow-up was analyzed. Exclusion criteria were age above 75 years, dialysis or renal transplantation, GFR < 30 ml/min/ 1.73 m^2^, nephrotic syndrome (proteinuria > 3 g/die), urinary tract infection or congestive heart failure. At baseline and follow-up a full clinical evaluation was performed according to a standard protocol, all biochemical analyses were performed in the same laboratory undergoing regular external quality control. BMI was calculated as weight (Kg) /height (m^2^). Supine blood pressure was measured with standard protocol. Medication use and smoking habits were assessed by interview.

Glycated haemoglobin (HbA1c) was measured by HPLC. Urinary albumin and creatinine were measured on a morning spot urine sample by immunonephelometry and enzymatic colorimetric methods, respectively; the albumin/creatinine ratio was calculated; values <30 μg/mg, 30–299 μg/mg and ≥300 μg/mg in the absence of haematuria were defined as normo, micro or macroalbuminuria. GFR was estimated with the Chronic Kidney Disease Epidemiology Collaboration (CKDEPI) Equation [21].

End points were UAER and estimated GFR. Progression of nephropathy was defined based on the occurrence of a composite end point including any of the followings : doubling of baseline UAER to at least 100 μg/mg; progression from normo to microalbuminuria or from micro to macroalbuminuria; decay of eGFR (i.e. 25% reduction from baseline, or an annualized eGFR decline >3 ml/min per year) [22,23].

Genomic DNA was isolated using Biorobot EZ1 Qiagen. By polymerase chain reaction (PCR) all samples were genotyped for the non-synonymous variation Pro12Ala (rs1801282) in the first exon of PPARγ2. The oligoprimers were tested by gradient PCR to optimize melting temperature. Genotyping was performed by an allele-specific amplification method using SYBR Green detection in a Realtime ABI PRISM 7000 apparatus (PE Applied Biosystem). In 5% of the samples genotyping was performed in duplicate and was fully concordant.

The study was approved by the university hospital ethics committee, informed consent was obtained from all participants.

### Statistical analysis

Data is given as mean ± standard deviation (SD) for normally distributed variables and as median and Interquartile range (IQR) for variables showing substantial asymmetry. Accordingly, groups were compared by unpaired or paired Student’s *t*-test, or by the equivalent non parametric procedures (Mann–Whitney and Wilcoxon matched pairs). Proportions were compared by Chi-square test. The Chi-square goodness-of-fit test was used to assess deviation from Hardy-Weinberg equilibrium of the genotypic frequency. The estimated proportions of subjects with a progression of nephropathy, as above defined, were derived with the use of the Kaplan-Meier product limit method and compared between groups by the Log-rank test. The Cox regression analysis was performed to explore the relationship between progression of nephropathy and Pro12Ala polymorphism after adjusting for potential confounding factors (i.e. age, diabetes duration, glucose control estimated by HbA1c, baseline UAER and eGFR, blood pressure and blockers of the renin angiotensin system. Results of the Cox regression model are reported as Hazard ratio (HR) with 95% Confidence Intervals (95% C.I.) All tests are two sided and p-value less than 0.05 was deemed as significant. The analyses were conducted using SPSS for Windows 16.0.

## Results

**Figure 1:**
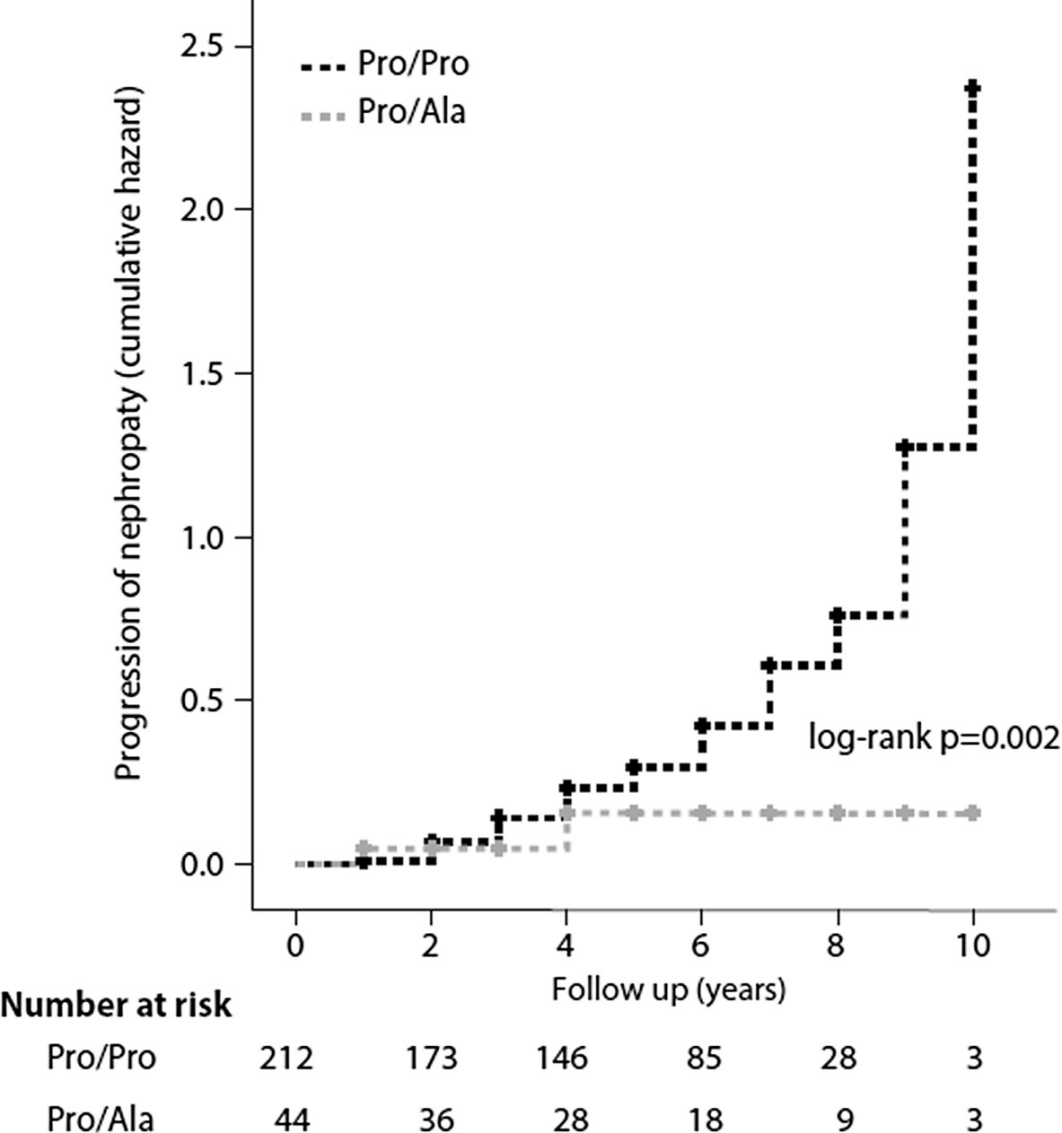
**Kaplan Meyer curves for progression of nephropathy (cumulative hazard) according to the Pro12Ala of PPARγ2 polymorphism.**

The genotype distribution was in Hardy Weinberg equilibrium, 212 participants (82.8%) were Pro/Pro homozygotes, 44 (17.2%) Pro/Ala heterozygotes, no homozygotes for the Ala variant were found. The characteristics of the study participants were similar in the two genotype groups. Age at onset of diabetes was 52.1 ± 9.6 and 53.6 ± 7.4 respectively in carriers or non carriers of the ala allele. Attained age, BMI, gender distribution, diabetes duration, glycated haemoglobin, blood pressure; UAER, eGFR and the proportion of people with micro/macroalbuminuria or GFR < 60 ml/min/ 1.73 m^2^, were comparable in the two genotype groups as was the proportion of current smokers, the proportion of people with retinopathy and the proportion of patients on antihypertensive medications (Table 1). As for antidiabetic treatment the proportion of people treated with diet, oral agents or insulin was similar in the two genotype groups. No one was treated with glitazones. The median follow-up was 5 years (IQR 3–7), similar in the two genotype groups. During the study period UAER remained stable in the Ala carriers [15.9 (IQR 9.4-26.3) versus 18.9 (IQR 11.9-30.7) μg/mg)], whereas a significant increase was observed in people with the ProPro genotype [17.0 (IQR 11.3-37.9 μg/mg) versus 24.5 (IQR 13.8-49.9) μg/mg], p = 0.006 (Table 2). The estimated GFR remained unchanged in Ala carriers (85.3 ± 12 vs 85.2 ± 13 ml/min/ 1.73 m2, p = ns) and declined slightly, but significantly, in the Pro/Pro group (82.8 ± 14.5 vs 80.3 ± 17.3 ml/min/ 1.73 m^2^, p =0.02) (Table 2). On average glucose control and blood pressure remained stable overtime in both genotype groups (Table 2). The proportion of patients with progression of nephropathy was significantly lower in patients carriers of the Ala polymorphism (11.4% vs 35.8%; p = 0.002, log rank test). The Cox regression analysis, performed with progression of nephropathy as the outcome variable and genotype, baseline age, UAER, eGFR, glycated haemoglobin, diabetes duration and blood pressure as predictor variables, confirmed a significant association of the Pro12Ala polymorphism with a lower risk of progression of nephropathy independent of established risk factors (HR = 0.32; 95% CI; 0.13-0.81 for12Ala carriers vs non carriers). A different model which included blockers of the renin angiotensin system instead of blood pressure provided similar results (HR = 0.30; 95% CI; 0.12-0.80). The Kaplan–Meier curve for progression of nephropathy in the two genotype groups is given in Figure 1.

**Table 1:** **Characteristics of the study population according to the Pro12Ala genotype**

	**Pro/Pro (N =212)**	**ProAla (N = 44)**
Males (%)	119 (56.1)	23 (52.3)
Age (years)	60.9 ± 4.1	60.3 ± 5.7
BMI (Kg/m^2^)	29.4 ± 4.0	29.6 ± 4.5
HB1Ac (%)	7.0 ± 1.5	6.9 ± 0.9
Systolic BP (mmHg)	135.3 ± 18.6	133.0 ± 27.6
Diastolic BP (mmHg)	78.4 ± 8.3	78.7 ± 15.9
Diabetes duration (years)	7.4 ± 6.3	8. 2 ± 7.3
Smokers (%)	49 (23.1)	11 (25)
With Rethinopathy (%)	49 (23.1)	12 (27.3)
Treated with diet (%)	20 (9.6)	5 (11.9)
Treated with oral agents (%)	138 (66)	29 (69.1)
Treated with insulin (%)	51 (24.4)	8 (19)
Treated with ACE or ARB (%)	155 (73.1)	29 (65.9)
Treated with Statins (%)	131 (61.8)	21 (47.7)
Microalbuminuria (%)	62 (29.2)	10 (22.7)
Macroalbuminuria (%)	8 (3.8)	2 (4.5)
GFR < 60 ml/min/ 1.73 m^2^ (%)	14 (6.7)	3 (7)

BP: Blood pressure UAER: Urinary albumin excretion rate; GFR: glomerular filtration rate.

**Table 2:** **Glomerular filtration rate (GFR), Urinary albumin excretion rate (UAER), glycemic control and blood pressure at baseline and follow-up according to genotype**

	**Pro/Pro**		**ProAla**	
	**Baseline**	**Follow-up**	**Baseline**	**Follow-up**
UAER (μg/mg)	17.1 (11.3-37.9)	24.5 (13.8-49.9)*	15.2 (9.4-26.3)	18.9 (11.9-30.7)
GFR (ml/min/ 1.73 m^2^)	82.8 ± 14.5	80.3 ± 17.3*	85.3. ±13.4	85.2 ± 13#
HbA1c (%)	7.0 ± 1.5	7.1 ± 1.4	6.9 ± 0.9	7.1 ± 0.8
PAS (mmHg)	135.3 ± 18.6	138.4 ± 22.5	133.0 ± 27.6	138.6 ± 23.1
PAD (mmHg)	78.4 ± 8.3	78.3 ± 10	78.7 ± 15.9	79.1 ± 12.6

*p < 0.05 vs baseline; # p < 0.05 vs Pro/Pro genotype.

## Discussion

The study conducted in a representative sample of patients with type 2 diabetes, shows that the Pro12Ala polymorphism of PPARγ2 is associated with a significantly lower progression of UAER, a significantly lower decay of GFR, significantly less overall renal damage,- as indicated by the lower incidence of a combined end point of clinically relevant progression of albuminuria and loss of renal function – as compared with carriers of the wild-type Pro/Pro genotype. To our knowledge this is the first study reporting on renal function overtime in relation to the Pro12Ala polymorphism of PPARγ2. Major risk factors for the progression of diabetic nephropathy such as diabetes duration, male sex and smoking habits were comparable in the two genotype groups, as well as age, baseline blood pressure, glucose control (HbA1c), UAER and eGFR, thus ruling out a confounding effect of these variables.

A lower progression of albumin excretion rate in carriers of the Ala allele is in line with the results of most, though not all, cross sectional studies, and recent meta-analyses reporting a lower prevalence of renal damage defined as increased UAER in carriers of the Ala allele [9-17] and is also coherent with results of the BENEDICT trial showing that in hypertensive diabetic patients with no evidence of renal injury at baseline, the Ala allele protects from new-onset microalbuminuria [20]. The present study expands current knowledge by providing data on progression to macroalbuminuria and decay of GFR over time which represent markers of more advanced stages of diabetic nephropathy. In addition the representativity of the study population makes the finding generalizable to the diabetic population at large.

To explore the mechanisms of the protection conferred by the Pro12Ala polymorphism of PPARγ2 is beyond the aims of the study and we can only speculate. Although PPARγ2 is generally perceived as a renoprotective factor in type 2 diabetes, how PPARγ*2* exerts its favourable effects remains unclear. Both systemic and organ-specific renoprotective effects of PPAR γ have been identified. At the systemic level PPARγ activation induces complex metabolic effects including improved insulin sensitivity, reduction of plasma glucose and blood pressure increased plasma adiponectin and reduced levels of circulating non esterified fatty acids (NEFA) and insulin-desensitizing cytokines [24].

Prior studies have suggested that carriers of the 12 Ala allele show significantly improved insulin sensitivity and protection from diabetes as compared with wild type Pro/Pro homozygotes [3,5]. In addition the Ala 12 variant has been associated with an increased resistance to oxidative stress resulting from overproduction of reactive oxygen species under hyperglycemic conditions [4]. Both insulin resistance and oxidative stress have been suggested as determinants of the development and progression of diabetic nephropathy.

Based on these evidence, one likely, and conceivably causal, mechanism through which the the alanine variant of PPARγ2 may exert renal protective effects is via reduction of insulin resistance and oxidative stress.

Furthermore multiple renal cell lines (glomerular mesangial cells, podocytes, tubular epithelium, microvasculature) have endogenous PPARγ expression and there is evidence that activation of PPARγ expressed in the kidney itself may provide additional benefits by reducing mesangial extracellular matrix production, maintaining podocyte number and function and limiting interstitial infiltration of monocytes/macrophages [25]. The hypothesis that the renoprotective properties of PPARγ may be partly independent of metabolic effects is further supported by studies showing that the PPARγ agonists thiazolidinendiones attenuate progression of renal damage in diabetic nepropathy as well as in nepropathies not associated with metabolic abnormalities, including toxic and immune mediated renal injury [26-28]. Whether the Ala variant impacts on the expression and activity of PPAR γ at the renal level is unexplored and it is worth investigating.

In conclusion results of this study show a differential susceptibility to progression of diabetic nephropathy and loss of renal function in carriers or non carriers of the Pro12Ala polymorphism of PPAR γ2. These results are coherent with current knowledge on the role of PPARγ on the pathophysiology of diabetic nephropathy and lend further support to a conceivably causal association of the Pro12 Ala polymorphism with protection from diabetic nephropathy.

For better interpreting the results, some study limitations should be acknowledged. Firstly the small sample size and the definition of albuminuria based on a single measurement limited the ability to draw more solid conclusions, both conditions, however, are likely to bias the findings towards null, rather than towards positive. Secondly, due the observational design the study is hypothesis generating rather than conclusive. Nonetheless this is the first report on protection from worsening of renal function overtime in carriers of the Pro12Ala polymorphism of PPAR γ2, in addition the selection of a study sample representative of the diabetic patients routinely seen in clinical practice makes the results generalizable to the diabetic population at large.
